# 376. Long Term Outcomes of a Pilot Single-Blinded, Randomized Controlled Trial Comparing BNT162b2 vs JNJ-78436735 Vaccine as the Third Dose after Two Doses of BNT162b2 Vaccine in Solid Organ Transplant Recipients

**DOI:** 10.1093/ofid/ofad500.446

**Published:** 2023-11-27

**Authors:** Yoichiro Natori, Eric F Martin, Adela Mattiazzi, Katherine Alexandra Sota, Julia Bini Viotti, Shweta Anjan, Giselle Guerra

**Affiliations:** University of Miami, Miami Transplant Institute, Jackson Health System, Miami, Florida; University of Miami Miller School of Medicine, Miami Transplant Institute, Miami, Florida; University of Miami, Miami, Florida; Miami Transplant Institute, Pembroke Pines, Florida; University of Miami/ Jackson Memorial Hospital, Miami, Florida; University of Miami Miller School of Medicine and Miami Transplant Institute, Jackson Health System, Miami, Florida; Miami Transplant Institute, Jackson Health System, Miami, Florida

## Abstract

**Background:**

Due to immunosuppressive medication, the immunogenicity after COVID-19 vaccination is suboptimal and durability in Solid Organ Transplant (SOT) recipients is still questionable. Thus, booster dose has been studied to improve not only the short but also long-term immunogenicity. However, mix and match method for this purpose was not well studied.

**Methods:**

This was a post hoc analysis of a patient-blinded, single center, randomized controlled trial comparing BNT162b2 vs JNJ-78436735 vaccine as the third dose after two doses of BNT162b2 vaccine. We included adult SOT recipients with functional graft who had received two doses of BNT162b2 vaccine and were randomly assigned to receive either vaccine. The outcome included SARS-CoV-2 IgG positivity at six months after the third dose and geometric mean titer.

**Results:**

4 SOT recipients, including 39 kidney, 3 liver, 1 lung, and 1 heart transplants, were analyzed per protocol. Only 1 recipient did not develop IgG after 6 months. Median IgG levels at 6 months were 5893 and 65584 for BNT162b2 vs JNJ-78436735, respectively(p< 0.01). For geometric mean titer from prior to third dose and 6 months after was 4.1 and 62.0 for BNT162b2 vs JNJ-78436735, respectively(p< 0.01).

**Conclusion:**

After 6 months post third dose, BNT162b2 showed significant higher immunogenicity in SOT recipients. BNT162b2 provided a higher IgG titer, and durability. To continue BNT162b2 vaccine boosters should be beneficial in SOT recipients but further study should be required.

**Disclosures:**

**Adela Mattiazzi, MD**, Care Dx: Advisor/Consultant|Care Dx: Speaker|Kaneka: Advisor/Consultant|Kaneka: Speaker

